# Crystal structure and Hirshfeld surface analysis of 3-(hy­droxy­meth­yl)-3-methyl-2,6-di­phenyl­piperidin-4-one

**DOI:** 10.1107/S2056989021012640

**Published:** 2022-01-01

**Authors:** Mustafa Kemal Gümüş, Sevgi Kansiz, Gulzhamal Bagitovna Tulemisova, Necmi Dege, Eiad Saif

**Affiliations:** a Science-Technology Research and Application Center, Artvin Coruh University, Artvin, Turkey; b Department of Chemistry and Chemical Technologies, Faculty of Natural and Agricultural Sciences, Atyrau State University named after Kh. Dosmukhamedov, 060011, Atyrau, Kazakhstan; c Samsun University, Faculty of Engineering, Department of Fundamental Sciences, 55420, Samsun, Turkey; d Ondokuz Mayıs University, Faculty of Arts and Sciences, Department of Physics, 55139, Samsun, Turkey; e Department of Computer and Electronic Engineering Technology, Sanaa Community College, Sanaa, Yemen; f Department of Electrical and Electronic Engineering, Faculty of Engineering, Ondokuz Mayıs University, 55139, Samsun, Turkey

**Keywords:** crystal structure, piperidone, benzaldehyde, 4-hy­droxy-3-methyl-2-butanone, ammonium acetate, Hirshfeld surface, hydrogen bonding

## Abstract

A new synthesis of the title compound, C_19_H_21_NO_2_, was developed with good yield and purity using the reaction of 4-hy­droxy-3-methyl-2-butanone, benzaldehyde and ammonium acetate in glacial acetic acid as a solvent. The central piperidine ring adopts a chair conformation, and its least-squares basal plane forms dihedral angles of 85.71 (11) and 77.27 (11)° with the terminal aromatic rings. In the crystal, the mol­ecules are linked by O—H⋯O and C—H⋯O hydrogen bonds into double ribbons. The Hirshfeld surface analysis shows that the most important contributions are from H⋯H (68%), C⋯H/H⋯C (19%) and O⋯H/H⋯O (12%) inter­actions.

## Abstract 2

3-(Hy­droxy­meth­yl)-3-methyl-2,6-di­phenyl­piperidin-4-one was synthesized by condensing 4-hy­droxy-3-methyl-2-butanone with the benzaldehyde and ammonium acetate. In the crystal, the mol­ecules are linked by O—H⋯O and C—H⋯O hydrogen bonds into double ribbons.

## Chemical context

Many piperidine derivatives are found to possess pharmacological activity and are constituents of important drugs. Numerous biological effects including anti­viral, anti­tumor, bactericidal, fungicidal and anti-inflammatory activities have been reported for these compounds (Kappe, 2000 ▸; Rameshkumar *et al.*, 2003 ▸; Sasitha & John, 2021 ▸). In this work, a new protocol for the synthesis of di­phenyl­piperidin-4-one from 4-hy­droxy-3-methyl-2-butanone, benzaldehyde and ammonium acetate under mild reaction conditions was developed. In addition, 3-(hy­droxy­meth­yl)-3-methyl-2,6-di­phenyl­piper­idin-4-one was characterized by single crystal X-ray diffraction and studied by Hirshfeld surface analysis. 
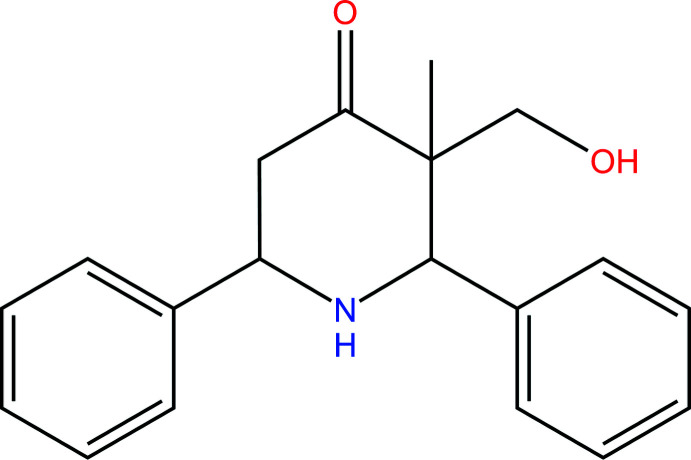


## Structural commentary

**Figure 1:**
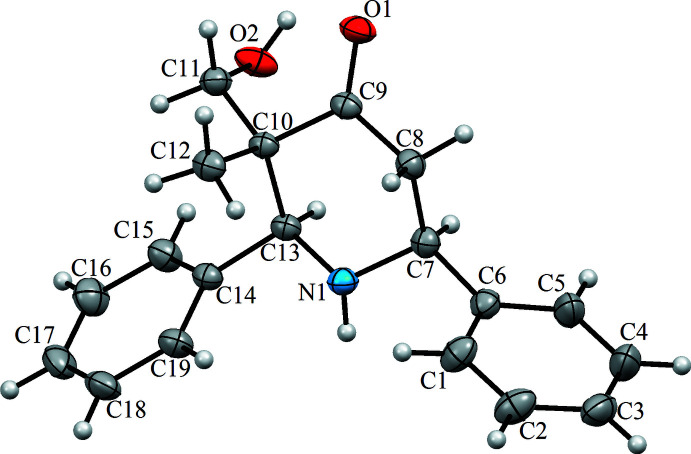
The mol­ecular structure of 3-(hy­droxy­meth­yl)-3-methyl-2,6-di­phenyl­piperidin-4-one with the atom labelling. Displacement ellipsoids are drawn at the 40% probability level.

The title compound, C_19_H_21_NO_2_, crystallizes in the space group *Pna*2_1_ with one mol­ecule in the asymmetric unit of the cell. As shown in Fig. 1 ▸, it involves two terminal aromatic rings (C1–C6 and C14–C19) and a central piperidinone fragment (N1/C7–C10/Cl3/O1). The piperidine ring adopts a chair conformation, with the carbonyl O1 and the N-bound H1 atoms being in the equatorial positions. The least-squares basal plane of the piperidine ring (C7, C8, C10, C13) makes dihedral angles of 85.71 (11) and 77.27 (11)°, respectively, with the planes of the C1–C6 and C14–C19 aromatic rings.

## Supra­molecular features

**Figure 2:**
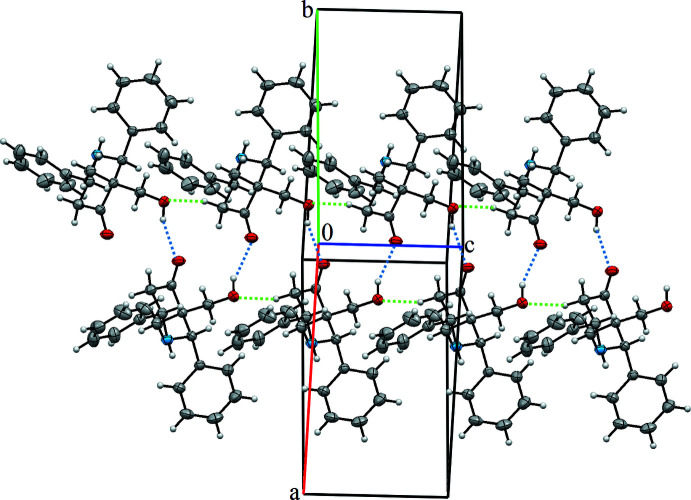
View of the hydrogen-bonded double ribbon in the title structure showing C8—H8*A*⋯O2 hydrogen bonds as green dashed lines and O2—H2⋯O1 hydrogen bonds as blue dashed lines.

In the crystal, mol­ecules of the title compound are linked by strong O—H⋯O and weak C—H⋯O hydrogen bonds (Table 1 ▸) into double ribbons stretched along the *c-*axis direction (Fig. 2 ▸). Neighbouring mol­ecules in the ribbon are related by the 2_1_ screw axis. Besides this, the mol­ecules are connected by N1—H1⋯C3 contacts into chains along the *b-*axis direction, thus layers perpendicular to the *a* axis are formed. No π–π or C—H⋯π inter­actions are present in this structure.

## Database survey

A search of the Cambridge Structural Database (CSD Version 5.42, update of May 2021; Groom *et al.*, 2016 ▸) revealed several related structures, *viz*. dimethyl-3-(2-hy­droxy­eth­yl)-9-oxo-7-phenyl­ethyl-6,8-diphenyl-3,7-di­aza­bicyclo­(3.3.1)nonane-1,5-di­carboxyl­ate (BACLUM; Caujolle *et al.*, 1981 ▸), dimethyl-3-methyl-2,4-bis­(4-nitro­phen­yl)-9-oxo-7-(1-phenyl­eth­yl)-3,7-di­aza­bicyclo­[3.3.1]nonane-1,5-di­carboxyl­ate (DEZTEK; Ros­setti *et al.*, 2018 ▸) and dimethyl-2,4-bis­(2-meth­oxy­phen­yl)-3,7-dimethyl-3,7-di­aza­bicyclo­(3.3.1)nonan-9-one-1,5-di­carboxyl­ate (REXNUD; Comba *et al.*, 1997 ▸). In these three structures, the piperidine rings adopt a chair conformation, as in the title compound.

## Hirshfeld surface analysis

**Figure 3:**
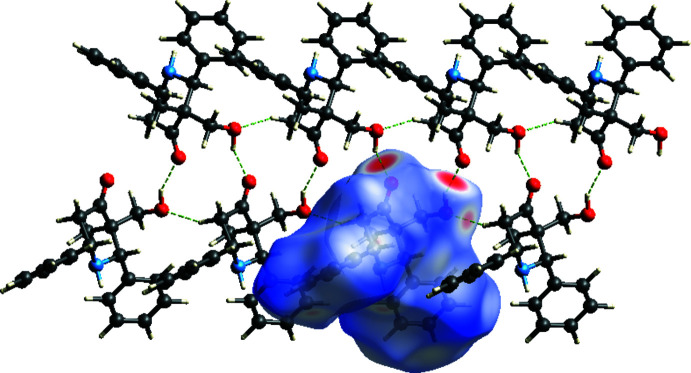
The red spots on the *d_norm_* surface of the title structure represent the O—H⋯O and C—H⋯O inter­molecular inter­actions.

**Figure 4:**
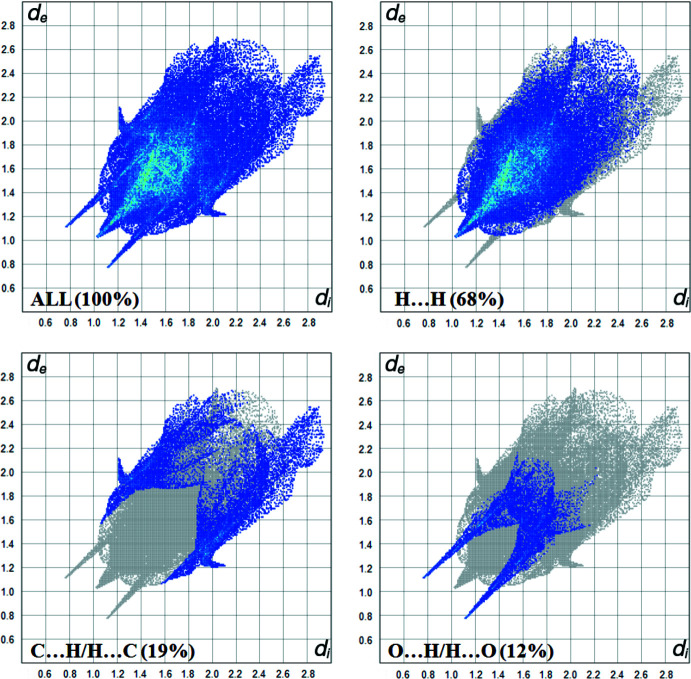
The view of the two-dimensional fingerprint plots for the title structure.

The Hirshfeld surface analysis of the title compound was performed using *Crystal Explorer 17* (Turner *et al.*, 2017 ▸; Spackman & Jayatilaka, 2009 ▸). Fig. 3 ▸ shows the 3D surface mapped over *d*_norm_ over the range −0.5456 (red) to 1.6913 (blue) a.u. The large and small red spots indicate the O—H⋯O and C—H⋯O inter­actions. The two-dimensional fingerprint plots, shown in Fig. 4 ▸, present all inter­actions and those delineated into H⋯H (68%), C⋯H/H⋯C (19%) and O⋯H/H⋯O (12%) components.

## Synthesis and crystallization

**Figure 5:**
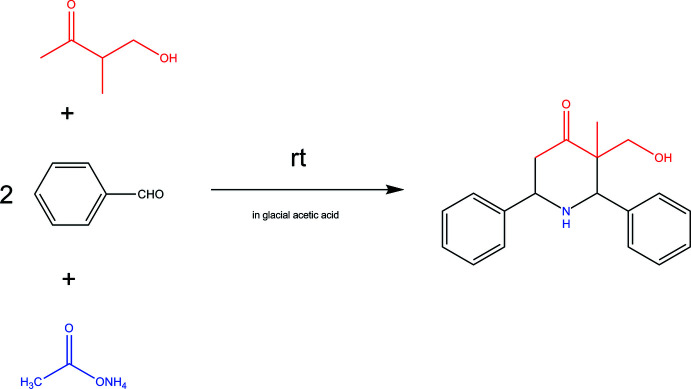
The synthesis of 3-(hy­droxy­meth­yl)-3-methyl-2,6-di­phenyl­piperidin-4-one.

The title compound was prepared (Fig. 5 ▸) according to the procedure reported in the literature for preparation of di­phenyl­piperidin-4-one (Kim & Tulemisova, 1997 ▸). To a mixture of 3.03 g (0.03 mol) of 4-hy­droxy-3-methyl-2-butanone and 6.04 g (0.06 mol) of benzaldehyde in glacial acetic acid as a solvent, kept at 293–298 K until the initial keto alcohol disappears as indicated by TLC (1.5 h), 2.3 g (0.03 mol) of ammonium acetate was added. Then the mixture was stirred at the same temperature for 6–7 h. The formed white precipitate was separated and after acidification of the solution with 5% hydro­chloric acid to pH 4, the hydro­chlorides were converted to bases by neutralization with K_2_CO_3_ in a strongly basic reaction. After the extraction with diethyl ether of the by-product base (control of the completeness of extraction by TLC), the title compound was extracted with chloro­form. After drying the chloro­form extracts and distilling off the solvent, a white crystalline compound was obtained (5.95 g, 70%), readily soluble in chloro­form, acetone, and hot ethanol (Fig. 5 ▸).

## Refinement

Crystal data, data collection and structure refinement details are summarized in Table 2 ▸. The N-bound H atom was refined freely. The O-bound H atom was located in a difference-Fourier map and refined with O—H = 0.82 Å, and with *U*_iso_(H) = 1.5*U*_eq_(O). The C-bound H atoms were positioned geometrically (C—H = 0.93, 0.96, 0.97 and 0.98 Å for *sp* ^2^-hybridized, methyl, methyl­ene and methine C atoms, respectively) and refined using a riding model, with *U*_iso_(H) = 1.5*U*_eq_(C) and 1.2*U*_eq_(C) for methyl and other H atoms, respectively.

## Supplementary Material

Crystal structure: contains datablock(s) I. DOI: 10.1107/S2056989021012640/yk2159sup1.cif

Structure factors: contains datablock(s) I. DOI: 10.1107/S2056989021012640/yk2159Isup2.hkl

Supporting information file. DOI: 10.1107/S2056989021012640/yk2159Isup3.cml

CCDC reference: 2124980

Additional supporting information:  crystallographic information; 3D view; checkCIF report

## Figures and Tables

**Table 1:** Hydrogen-bond geometry (Å, °)

*D*—H⋯*A*	*D*—H	H⋯*A*	*D*⋯*A*	*D*—H⋯*A*
O2—H2⋯O1^i^	0.82	2.05	2.8194 (18)	156
C8—H8*A*⋯O2^ii^	0.97	2.47	3.379 (3)	155
N1—H1⋯C3^iii^	0.90 (3)	2.75 (3)	3.605 (2)	161 (3)

Symmetry codes: (i) -x-1, -y-1, z+{\script{1\over 2}}; (ii) x, y, z-1; (iii) -x-1, -y, z+{\script{1\over 2}}.

**Table 2:** Experimental details

Crystal data	
Chemical formula	C_19_H_21_NO_2_
*M*_r_	295.37
Crystal system, space group	Orthorhombic, *P* *n* *a*2_1_
Temperature (K)	296
*a*, *b*, *c* (Å)	17.3298 (8), 14.1856 (7), 6.5857 (3)
*V* (Å^3^)	1618.99 (13)
*Z*	4
Radiation type	Mo *K*α
μ (mm^−1^)	0.08
Crystal size (mm)	0.72 × 0.57 × 0.33

Data collection	
Diffractometer	Stoe IPDS 2
Absorption correction	Integration (*X-RED32*; Stoe & Cie, 2002 ▸)
*T*_min_, *T*_max_	0.958, 0.973
No. of measured, independent and observed [*I* > 2σ(*I*)] reflections	17314, 4763, 3441
*R*_int_	0.042
(sin θ/λ)_max_ (Å^−1^)	0.729

Refinement	
*R*[*F* ^2^ > 2σ(*F* ^2^)], *wR*(*F* ^2^), *S*	0.041, 0.096, 1.01
No. of reflections	4763
No. of parameters	204
No. of restraints	1
H-atom treatment	H atoms treated by a mixture of independent and constrained refinement
Δρ_max_, Δρ_min_ (e Å^−3^)	0.16, −0.19
Absolute structure	Flack *x* determined using 1072 quotients [(*I* ^+^)−(*I* ^−^)]/[(*I* ^+^)+(*I* ^−^)] (Parsons et al., 2013 ▸)
Absolute structure parameter	0.8 (5)

Computer programs: *X-AREA* and *X-RED* (Stoe & Cie, 2002 ▸), *SHELXT2017/1* (Sheldrick, 2015*a* ▸), *SHELXL2017/1* (Sheldrick, 2015*b* ▸), *PLATON* (Spek, 2020 ▸), *WinGX* (Farrugia, 2012 ▸) and *publCIF* (Westrip, 2010 ▸).

## supplementary crystallographic information

### Crystal data

**Table:**

C_19_H_21_NO_2_	*D*_x_ = 1.212 Mg m^−^^3^
*M**_r_* = 295.37	Mo *K*α radiation, λ = 0.71073 Å
Orthorhombic, *P**n**a*2_1_	Cell parameters from 17006 reflections
*a* = 17.3298 (8) Å	θ = 1.9–31.5°
*b* = 14.1856 (7) Å	µ = 0.08 mm^−^^1^
*c* = 6.5857 (3) Å	*T* = 296 K
*V* = 1618.99 (13) Å^3^	Prism, colorless
*Z* = 4	0.72 × 0.57 × 0.33 mm
*F*(000) = 632	

### Data collection

**Table:**

Stoe IPDS 2 diffractometer	4763 independent reflections
Radiation source: sealed X-ray tube, 12 x 0.4 mm long-fine focus	3441 reflections with *I* > 2σ(*I*)
Detector resolution: 6.67 pixels mm^-1^	*R*_int_ = 0.042
rotation method scans	θ_max_ = 31.2°, θ_min_ = 1.9°
Absorption correction: integration (X-RED32; Stoe & Cie, 2002)	*h* = −24→25
*T*_min_ = 0.958, *T*_max_ = 0.973	*k* = −20→20
17314 measured reflections	*l* = −7→9

### Refinement

**Table:**

Refinement on *F*^2^	Secondary atom site location: difference Fourier map
Least-squares matrix: full	Hydrogen site location: mixed
*R*[*F*^2^ > 2σ(*F*^2^)] = 0.041	H atoms treated by a mixture of independent and constrained refinement
*wR*(*F*^2^) = 0.096	*w* = 1/[σ^2^(*F*_o_^2^) + (0.0515*P*)^2^] where *P* = (*F*_o_^2^ + 2*F*_c_^2^)/3
*S* = 1.01	(Δ/σ)_max_ < 0.001
4763 reflections	Δρ_max_ = 0.16 e Å^−^^3^
204 parameters	Δρ_min_ = −0.19 e Å^−^^3^
1 restraint	Absolute structure: Flack *x* determined using 1072 quotients [(*I*^+^)-(*I*^-^)]/[(*I*^+^)+(*I*^-^)] (Parsons et al., 2013)
Primary atom site location: structure-invariant direct methods	Absolute structure parameter: 0.8 (5)

### Special details

**Table:**

Geometry. All esds (except the esd in the dihedral angle between two l.s. planes) are estimated using the full covariance matrix. The cell esds are taken into account individually in the estimation of esds in distances, angles and torsion angles; correlations between esds in cell parameters are only used when they are defined by crystal symmetry. An approximate (isotropic) treatment of cell esds is used for estimating esds involving l.s. planes.

### Fractional atomic coordinates and isotropic or equivalent isotropic displacement parameters (Å^2^)

**Table:**

	*x*	*y*	*z*	*U*_iso_*/*U*_eq_	
O1	−0.52068 (8)	−0.46901 (9)	−0.4103 (2)	0.0600 (4)	
O2	−0.60074 (9)	−0.40945 (10)	−0.0228 (2)	0.0610 (4)	
H2	−0.565229	−0.447609	−0.026638	0.091*	
N1	−0.58527 (9)	−0.20075 (10)	−0.4826 (2)	0.0446 (3)	
C14	−0.69489 (10)	−0.21260 (11)	−0.2492 (3)	0.0421 (4)	
C9	−0.54765 (10)	−0.39351 (11)	−0.4587 (3)	0.0417 (4)	
C13	−0.61836 (10)	−0.25456 (10)	−0.3131 (3)	0.0396 (3)	
H13	−0.582802	−0.250220	−0.197803	0.048*	
C6	−0.47125 (11)	−0.16339 (12)	−0.6872 (3)	0.0483 (4)	
C10	−0.62508 (9)	−0.36075 (11)	−0.3756 (3)	0.0388 (3)	
C11	−0.64790 (12)	−0.42148 (12)	−0.1929 (3)	0.0482 (4)	
H11A	−0.700646	−0.406616	−0.155468	0.058*	
H11B	−0.646451	−0.487258	−0.233009	0.058*	
C7	−0.50597 (10)	−0.22790 (12)	−0.5292 (3)	0.0453 (4)	
H7	−0.474939	−0.224784	−0.404983	0.054*	
C19	−0.74209 (11)	−0.16475 (13)	−0.3861 (3)	0.0515 (4)	
H19	−0.725854	−0.156467	−0.519485	0.062*	
C8	−0.50769 (11)	−0.32968 (13)	−0.6060 (3)	0.0484 (4)	
H8A	−0.534221	−0.331932	−0.735585	0.058*	
H8B	−0.455272	−0.351604	−0.626845	0.058*	
C12	−0.68510 (11)	−0.37561 (13)	−0.5433 (3)	0.0508 (4)	
H12A	−0.674874	−0.333097	−0.653506	0.076*	
H12B	−0.735741	−0.363652	−0.490172	0.076*	
H12C	−0.682287	−0.439401	−0.591446	0.076*	
C15	−0.72036 (12)	−0.22177 (14)	−0.0520 (3)	0.0555 (5)	
H15	−0.688985	−0.251458	0.043011	0.067*	
C18	−0.81260 (13)	−0.12956 (14)	−0.3257 (4)	0.0638 (6)	
H18	−0.843205	−0.097262	−0.418500	0.077*	
C1	−0.51207 (14)	−0.13749 (15)	−0.8595 (4)	0.0630 (5)	
H1A	−0.562927	−0.157131	−0.875089	0.076*	
C17	−0.83831 (13)	−0.14174 (16)	−0.1288 (4)	0.0692 (6)	
H17	−0.886403	−0.119157	−0.089324	0.083*	
C3	−0.40316 (15)	−0.05295 (15)	−0.9865 (4)	0.0730 (7)	
H3	−0.379964	−0.017017	−1.087645	0.088*	
C16	−0.79174 (14)	−0.18768 (16)	0.0075 (4)	0.0687 (6)	
H16	−0.808210	−0.195995	0.140665	0.082*	
C2	−0.47809 (16)	−0.08286 (16)	−1.0084 (4)	0.0717 (6)	
H2A	−0.505992	−0.066302	−1.123614	0.086*	
C5	−0.39677 (12)	−0.13063 (17)	−0.6673 (5)	0.0715 (7)	
H5	−0.368693	−0.145511	−0.551306	0.086*	
C4	−0.36317 (14)	−0.07616 (19)	−0.8164 (5)	0.0847 (9)	
H4	−0.312707	−0.055149	−0.800405	0.102*	
H1	−0.5902 (17)	−0.140 (2)	−0.450 (5)	0.102*	

### Atomic displacement parameters (Å^2^)

**Table:**

	*U*^11^	*U*^22^	*U*^33^	*U*^12^	*U*^13^	*U*^23^
O1	0.0654 (9)	0.0459 (7)	0.0686 (9)	0.0199 (6)	0.0128 (8)	0.0080 (6)
O2	0.0811 (10)	0.0618 (7)	0.0400 (7)	0.0271 (7)	−0.0050 (7)	0.0015 (6)
N1	0.0489 (8)	0.0368 (6)	0.0480 (9)	0.0024 (6)	0.0031 (7)	0.0004 (6)
C14	0.0454 (9)	0.0369 (7)	0.0439 (9)	0.0037 (7)	−0.0020 (8)	−0.0021 (6)
C9	0.0468 (9)	0.0383 (7)	0.0399 (8)	0.0044 (7)	−0.0039 (7)	−0.0052 (6)
C13	0.0428 (9)	0.0376 (7)	0.0383 (8)	0.0038 (6)	−0.0031 (7)	0.0005 (7)
C6	0.0484 (10)	0.0428 (8)	0.0537 (11)	−0.0033 (7)	0.0038 (9)	−0.0028 (8)
C10	0.0424 (8)	0.0370 (7)	0.0372 (8)	0.0030 (6)	−0.0040 (7)	0.0002 (6)
C11	0.0524 (10)	0.0425 (8)	0.0495 (10)	0.0046 (7)	0.0047 (9)	0.0050 (7)
C7	0.0448 (9)	0.0468 (8)	0.0441 (9)	−0.0011 (7)	−0.0013 (8)	−0.0006 (8)
C19	0.0549 (11)	0.0475 (9)	0.0521 (10)	0.0092 (8)	−0.0043 (9)	0.0034 (8)
C8	0.0478 (10)	0.0465 (9)	0.0509 (10)	0.0044 (8)	0.0061 (8)	−0.0008 (8)
C12	0.0536 (11)	0.0482 (9)	0.0507 (10)	0.0017 (8)	−0.0117 (9)	−0.0064 (8)
C15	0.0594 (11)	0.0600 (10)	0.0471 (11)	0.0121 (9)	0.0023 (9)	0.0022 (9)
C18	0.0584 (12)	0.0527 (10)	0.0803 (16)	0.0175 (9)	−0.0110 (11)	0.0034 (11)
C1	0.0735 (14)	0.0611 (11)	0.0544 (12)	−0.0208 (10)	−0.0075 (11)	0.0033 (10)
C17	0.0518 (12)	0.0604 (11)	0.0954 (19)	0.0141 (10)	0.0101 (13)	−0.0121 (12)
C3	0.0806 (16)	0.0533 (10)	0.0851 (18)	0.0009 (10)	0.0272 (14)	0.0154 (12)
C16	0.0682 (13)	0.0748 (13)	0.0632 (14)	0.0105 (11)	0.0176 (12)	−0.0050 (11)
C2	0.0980 (17)	0.0631 (12)	0.0540 (13)	−0.0150 (12)	−0.0030 (12)	0.0060 (11)
C5	0.0459 (12)	0.0746 (14)	0.0940 (19)	−0.0035 (10)	−0.0049 (12)	0.0283 (14)
C4	0.0533 (13)	0.0824 (16)	0.119 (3)	−0.0099 (11)	0.0055 (15)	0.0365 (17)

### Geometric parameters (Å, º)

**Table:**

O1—C9	1.211 (2)	C19—H19	0.9300
O2—C11	1.397 (2)	C8—H8A	0.9700
O2—H2	0.8200	C8—H8B	0.9700
N1—C7	1.460 (2)	C12—H12A	0.9600
N1—C13	1.469 (2)	C12—H12B	0.9600
N1—H1	0.90 (3)	C12—H12C	0.9600
C14—C15	1.378 (3)	C15—C16	1.385 (3)
C14—C19	1.394 (3)	C15—H15	0.9300
C14—C13	1.513 (2)	C18—C17	1.382 (4)
C9—C8	1.497 (3)	C18—H18	0.9300
C9—C10	1.522 (2)	C1—C2	1.381 (3)
C13—C10	1.566 (2)	C1—H1A	0.9300
C13—H13	0.9800	C17—C16	1.372 (4)
C6—C5	1.378 (3)	C17—H17	0.9300
C6—C1	1.387 (3)	C3—C4	1.358 (4)
C6—C7	1.511 (3)	C3—C2	1.374 (4)
C10—C12	1.532 (3)	C3—H3	0.9300
C10—C11	1.531 (2)	C16—H16	0.9300
C11—H11A	0.9700	C2—H2A	0.9300
C11—H11B	0.9700	C5—C4	1.379 (4)
C7—C8	1.530 (3)	C5—H5	0.9300
C7—H7	0.9800	C4—H4	0.9300
C19—C18	1.379 (3)		
			
C11—O2—H2	109.5	C9—C8—C7	111.45 (15)
C7—N1—C13	112.98 (14)	C9—C8—H8A	109.3
C7—N1—H1	113.2 (19)	C7—C8—H8A	109.3
C13—N1—H1	107 (2)	C9—C8—H8B	109.3
C15—C14—C19	117.87 (17)	C7—C8—H8B	109.3
C15—C14—C13	120.38 (16)	H8A—C8—H8B	108.0
C19—C14—C13	121.74 (17)	C10—C12—H12A	109.5
O1—C9—C8	121.79 (16)	C10—C12—H12B	109.5
O1—C9—C10	121.04 (16)	H12A—C12—H12B	109.5
C8—C9—C10	117.15 (14)	C10—C12—H12C	109.5
N1—C13—C14	110.43 (13)	H12A—C12—H12C	109.5
N1—C13—C10	109.24 (13)	H12B—C12—H12C	109.5
C14—C13—C10	112.72 (13)	C14—C15—C16	121.3 (2)
N1—C13—H13	108.1	C14—C15—H15	119.3
C14—C13—H13	108.1	C16—C15—H15	119.3
C10—C13—H13	108.1	C19—C18—C17	120.7 (2)
C5—C6—C1	117.8 (2)	C19—C18—H18	119.6
C5—C6—C7	120.78 (19)	C17—C18—H18	119.6
C1—C6—C7	121.40 (17)	C2—C1—C6	120.8 (2)
C9—C10—C12	107.29 (14)	C2—C1—H1A	119.6
C9—C10—C11	109.78 (14)	C6—C1—H1A	119.6
C12—C10—C11	108.28 (15)	C16—C17—C18	118.9 (2)
C9—C10—C13	108.82 (13)	C16—C17—H17	120.5
C12—C10—C13	111.86 (13)	C18—C17—H17	120.5
C11—C10—C13	110.75 (14)	C4—C3—C2	119.6 (2)
O2—C11—C10	114.21 (15)	C4—C3—H3	120.2
O2—C11—H11A	108.7	C2—C3—H3	120.2
C10—C11—H11A	108.7	C17—C16—C15	120.4 (2)
O2—C11—H11B	108.7	C17—C16—H16	119.8
C10—C11—H11B	108.7	C15—C16—H16	119.8
H11A—C11—H11B	107.6	C3—C2—C1	120.1 (3)
N1—C7—C6	111.10 (15)	C3—C2—H2A	119.9
N1—C7—C8	107.46 (14)	C1—C2—H2A	119.9
C6—C7—C8	110.59 (16)	C4—C5—C6	121.1 (2)
N1—C7—H7	109.2	C4—C5—H5	119.4
C6—C7—H7	109.2	C6—C5—H5	119.4
C8—C7—H7	109.2	C3—C4—C5	120.5 (2)
C18—C19—C14	120.7 (2)	C3—C4—H4	119.7
C18—C19—H19	119.7	C5—C4—H4	119.7
C14—C19—H19	119.7		
			
C7—N1—C13—C14	170.51 (14)	C1—C6—C7—N1	−44.8 (2)
C7—N1—C13—C10	−64.98 (17)	C5—C6—C7—C8	−103.5 (2)
C15—C14—C13—N1	−152.01 (17)	C1—C6—C7—C8	74.5 (2)
C19—C14—C13—N1	28.7 (2)	C15—C14—C19—C18	−1.2 (3)
C15—C14—C13—C10	85.5 (2)	C13—C14—C19—C18	178.07 (17)
C19—C14—C13—C10	−93.80 (19)	O1—C9—C8—C7	−133.78 (19)
O1—C9—C10—C12	−102.5 (2)	C10—C9—C8—C7	47.8 (2)
C8—C9—C10—C12	75.99 (19)	N1—C7—C8—C9	−54.0 (2)
O1—C9—C10—C11	15.0 (2)	C6—C7—C8—C9	−175.41 (15)
C8—C9—C10—C11	−166.55 (15)	C19—C14—C15—C16	2.3 (3)
O1—C9—C10—C13	136.33 (17)	C13—C14—C15—C16	−177.03 (19)
C8—C9—C10—C13	−45.2 (2)	C14—C19—C18—C17	−0.6 (3)
N1—C13—C10—C9	50.76 (17)	C5—C6—C1—C2	1.8 (3)
C14—C13—C10—C9	173.93 (15)	C7—C6—C1—C2	−176.3 (2)
N1—C13—C10—C12	−67.59 (18)	C19—C18—C17—C16	1.4 (4)
C14—C13—C10—C12	55.57 (19)	C18—C17—C16—C15	−0.4 (4)
N1—C13—C10—C11	171.52 (14)	C14—C15—C16—C17	−1.5 (3)
C14—C13—C10—C11	−65.32 (18)	C4—C3—C2—C1	−1.0 (4)
C9—C10—C11—O2	67.49 (18)	C6—C1—C2—C3	−0.4 (4)
C12—C10—C11—O2	−175.68 (14)	C1—C6—C5—C4	−1.8 (4)
C13—C10—C11—O2	−52.70 (19)	C7—C6—C5—C4	176.2 (2)
C13—N1—C7—C6	−173.39 (15)	C2—C3—C4—C5	1.0 (4)
C13—N1—C7—C8	65.51 (18)	C6—C5—C4—C3	0.5 (4)
C5—C6—C7—N1	137.2 (2)		

### Hydrogen-bond geometry (Å, º)

**Table:**

*D*—H···*A*	*D*—H	H···*A*	*D*···*A*	*D*—H···*A*
O2—H2···O1^i^	0.82	2.05	2.8194 (18)	156
C8—H8*A*···O2^ii^	0.97	2.47	3.379 (3)	155
N1—H1···C3^iii^	0.90 (3)	2.75 (3)	3.605 (2)	161 (3)

Symmetry codes: (i) −*x*−1, −*y*−1, *z*+1/2; (ii) *x*, *y*, *z*−1; (iii) −*x*−1, −*y*, *z*+1/2.
